# The TT Genotype of the *STAT4* rs7574865 Polymorphism Is Associated with High Disease Activity and Disability in Patients with Early Arthritis

**DOI:** 10.1371/journal.pone.0043661

**Published:** 2012-08-24

**Authors:** Amalia Lamana, Alejandro Balsa, Blanca Rueda, Ana M. Ortiz, Laura Nuño, Maria Eugenia Miranda-Carus, Maria F. Gonzalez-Escribano, Miguel A. Lopez-Nevot, Dora Pascual-Salcedo, Javier Martin, Isidoro González-Álvaro

**Affiliations:** 1 Rheumatology Service, Hospital Universitario de La Princesa, Instituto de Investigación Sanitaria Princesa, Madrid, Spain; 2 Rheumatology Service, Hospital Universitario La Paz, IdiPaz, Instituto de Investigación Sanitaria La Paz, Madrid, Spain; 3 Departamento de Enfermería, Facultad de Ciencias de la Salud, Universidad de Granada, Granada, Spain; 4 Immunology Service, Hospital Universitario Virgen del Rocio, Sevilla, Spain; 5 Immunology Service, Hospital Universitario Virgen de las Nieves, Granada, Spain; 6 Immunology Service, Hospital Universitario La Paz, IdiPaz, Instituto de Investigación Sanitaria La Paz, Madrid, Spain; 7 Instituto de Parasitología y Biomedicina Lopez Neyra, CSIC, Granada, Spain; University of Michigan, United States of America

## Abstract

**Background:**

The number of copies of the *HLA-DRB1* shared epitope, and the minor alleles of the *STAT4* rs7574865 and the *PTPN22* rs2476601 polymorphisms have all been linked with an increased risk of developing rheumatoid arthritis. In the present study, we investigated the effects of these genetic variants on disease activity and disability in patients with early arthritis.

**Methodology and Results:**

We studied 640 patients with early arthritis (76% women; median age, 52 years), recording disease-related variables every 6 months during a 2-year follow-up. *HLA-DRB1* alleles were determined by PCR-SSO, while rs7574865 and rs2476601 were genotyped with the Taqman 5′ allelic discrimination assay. Multivariate analysis was performed using generalized estimating equations for repeated measures. After adjusting for confounding variables such as gender, age and ACPA, the TT genotype of rs7574865 in *STAT4* was associated with increased disease activity (DAS28) as compared with the GG genotype (β coefficient [95% confidence interval] = 0.42 [0.01–0.83], p = 0.044). Conversely, the presence of the T allele of rs2476601 in *PTPN22* was associated with diminished disease activity during follow-up in a dose-dependent manner (CT genotype = −0.27 [−0.56– −0.01], p = 0.042; TT genotype = −0.68 [−1.64– −0.27], p = 0.162). After adjustment for gender, age and disease activity, homozygosity for the T allele of rs7574865 in *STAT4* was associated with greater disability as compared with the GG genotype.

**Conclusions:**

Our data suggest that patients with early arthritis who are homozygous for the T allele of rs7574865 in *STAT4* may develop a more severe form of the disease with increased disease activity and disability.

## Introduction

Rheumatoid arthritis (RA) is caused by an interaction between genetic and environmental factors that leads to a break in immunological self-tolerance. Twin concordance data suggest that RA has a heritability rate of 50–60% [1]. The HLA region is the main genetic factor accounting for this heritability, particularly the *HLA-DRB1* alleles that encode the shared epitope (SE) [2]. In addition, during the past few years, genome-wide association studies and candidate gene association studies have led to the identification of new genes associated with the risk of developing RA [3]. The role of *PTPN22* and *STAT4* as genetic markers of susceptibility to RA has been extensively replicated in different populations [3]–[5].

In contrast to the clear association between the risk of developing RA and alleles in *HLA-DRB1*, *PTPN22*, and *STAT4*, it remains unclear whether these genetic factors affect disease severity once immunological self-tolerance is broken. Some authors have reported an association between the SE and radiological progression, systemic complications and cardiovascular events in RA [6]–[9]. However, several studies of the role of genetic factors outside the HLA region in the radiological progression of RA have produced contradictory results [10], and little information is available on the impact of genetic factors on disease activity and disability [11].

When our study was designed, only the presence of the SE and the polymorphisms in *PTPN22* and *STAT4* were clearly associated with an increased risk of developing RA in distinct Spanish populations. Thus, the objective of our study was to determine whether the presence of these genetic markers contributes to a poorer disease course and increased disability in patients with early arthritis (EA).

**Table 1:** Baseline characteristics of patients with early arthritis.

	Rheumatoid arthritis (n = 464)	Undifferentiated arthritis(n = 177)	Total (n = 641)	p value
Age (years)	53 (42–68)	51 (38–65)	52 (41–66)	0.022
Female gender (%)	75.6	76.8	75.9	NS
Disease duration(weeks)	18 (10–26)	14 (7–28)	17 (8–27)	0.039
Smokers (%)	43.9	40.2	42.9	NS
DAS28-ESR	5.4 (4.4–6.4)	3.8 (2.9–4.7)	5 (3.8–6.1)	<0.001
HAQ	1.250 (0.750–1.875)	0.875 (0.375–1.375)	1.125 (0.625–1.750)	<0.001
CRP (mg/dl)	1.2 (0.5–3.1)	0.5 (0.3–1.2)	0.9 (0.3–2.7)	<0.001
ESR (mm/h)	32 (20–56)	20 (12–33)	28 (17–50)	<0.001
ACPA-positive (%)	61.2	18.4	49.4	<0.001
RF positive (%)	67.7	16.9	53.7	<0.001
Shared epitope (%)	59.2	35.9	52.6	<0.001
*STAT4* (%) (GG – GT – TT)	58–34.7–7.3	59–35.9–5.1	58.2–35–6.7	NS
*PTPN22* (%) (CC – CT – TT)	83.3–15.7–0.9	86.4–13–0.6	84.1–15–0.9	NS

DAS28-ESR, 28-joint Disease Activity Score; HAQ, Health Assessment Questionnaire; CRP, C-reactive protein; ESR, erythrocyte sedimentation rate; ACPA, anti-citrullinated peptide antibodies; RF, rheumatoid factor; NS, non-significant.

## Methods

### Ethics Statement

The local ethics committees reviewed and approved the protocols of both EA Clinical Registers, and all the patients signed an informed consent form prior to inclusion in the study.

### Objective

The hypothesis of the present study is that the genetic variants that confer a high risk of developing RA are also associated with a worse clinical course of disease. The specific objective was to determine whether the presence of the SE, the minor alleles of *STAT4* rs7574865 or *PTPN22* rs2476601 polymorphisms affect disease activity and disability in EA patients.

**Figure 1:**
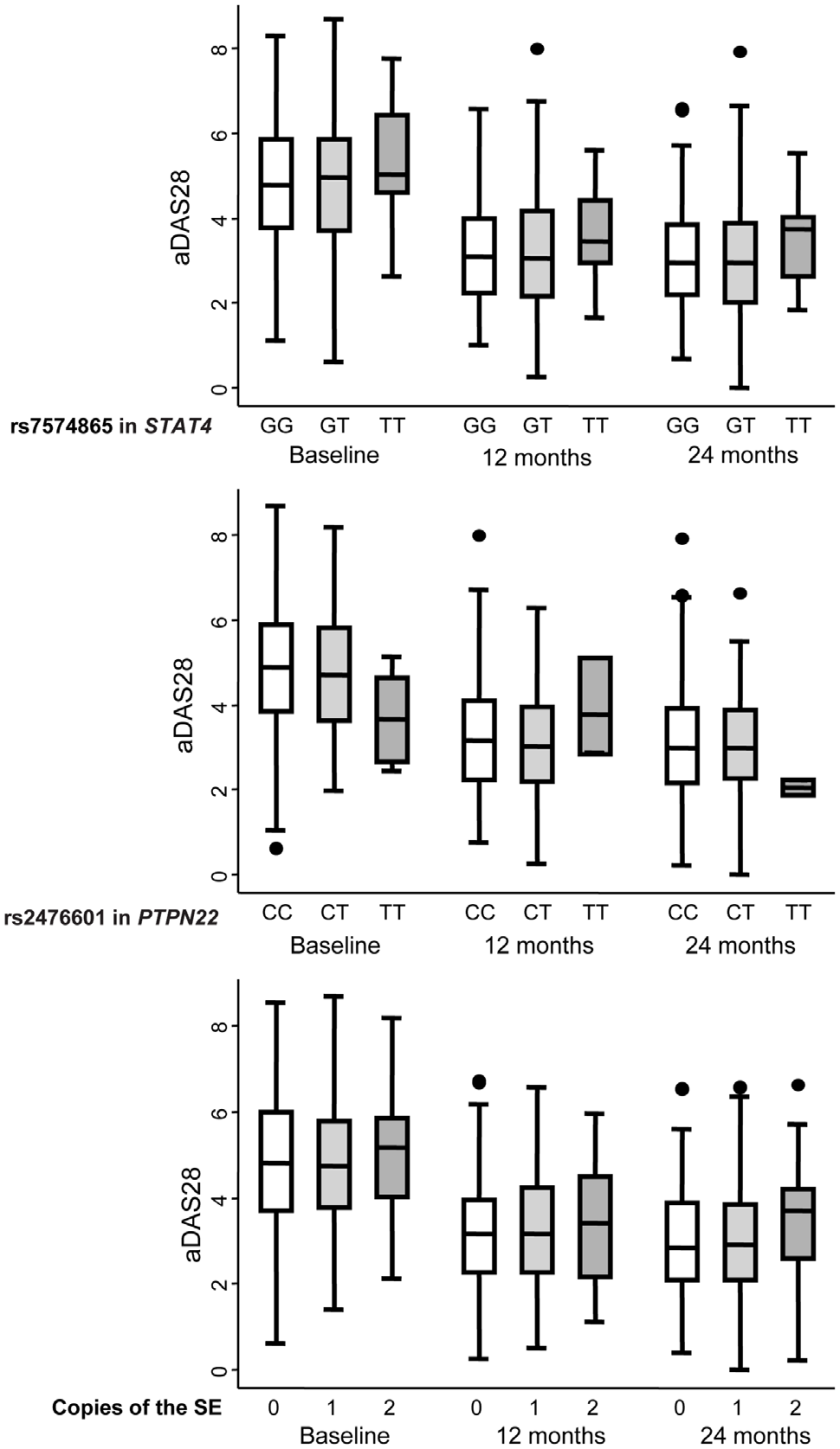
Patients with early arthritis who were homozygous for the minor rs7574865 allele of *STAT4* exhibited stronger disease activity during the follow-up. The data are categorized according to the rs7574865 genotype of *STAT4* (upper panel), the rs2476601 genotype of *PTPN22* (middle panel), and the number of copies of the shared epitope (SE; lower panel). Data represent the interquartile range (p75, upper edge of the box; p25, lower edge; p50, midline), p95 (line above the box) and p5 (line below the box) of the DAS28 adjusted for gender and age (aDAS28). Dots represent outliers.

**Table 2:** Effect of the presence of the shared epitope and the minor rs7574865 allele in *STAT4* or rs2476601 in *PTPN22* on disease activity (DAS28) and disability (HAQ) in patients with early arthritis.

	DAS28		HAQ	
	β Coeff. (95% CI)	p value	β Coeff. (95% CI)	p value
Age (years):				
<45	Ref.		Ref.	
45–65	0.54 (0.31–0.78)	<0.001	0.06 (−0.01–0.14)	0.108
>65	0.71 (0.46–0.97)	<0.001	0.11 (0.02–0.19)	0.011
Gender				
Man	Ref.		Ref.	
Woman	0.48 (0.31–0.78)	<0.001	0.09 (0.02–0.17)	0.016
Diagnosis				
RA	Ref.		Ref.	
UA	−0.50 (−0.81– −0.20)	0.001	−0.10 (−0.19− −0.01)	0.023
Positive ACPA	0.21 (0.01–0.42)	0.049	NS	NS
DAS28	NI	NI	0.30 (0.28–0.31)	<0.001
Shared epitope	NS	NS	NS	NS
*STAT4* (rs7574865)				
GG	Ref.		Ref.	
GT	−0.01 (−0.21–0.20)	0.953	0.07 (0.01–0.14)	0.055
TT	0.42 (0.01–0.83)	0.044	0.19 (0.05–0.32)	0.007
*PTPN22* (rs2476601)				
CC	Ref.		Ref.	
CT	−0.27 (−0.56– −0.01)	0.042	NS	NS
TT	−0.68 (−1.64–0.27)	0.162	NS	NS
Hospital				
Center 1	Ref.		Ref.	
Center 2	−0.35 (−0.56– −0.13)	<0.001	NS	NS

DAS28, 28-joint Disease Activity Score; HAQ, Health Assessment Questionnaire; Coeff., coefficient; Ref., reference; RA, rheumatoid arthritis; UA, undifferentiated arthritis; ACPA, anti-citrullinated peptide antibodies; NI, not included; NS, non-significant.

### Participants

The study sample comprised 641 patients with EA who attended the Hospital Universitario (HU) La Paz (355 patients) and HU La Princesa (286 patients). Inclusion criteria included 2 or more swollen joints for at least 4 weeks and symptoms for less than a year. Only data from patients fulfilling the 1987 American College of Rheumatology criteria for RA [12] within the 2-year follow-up (n = 464) or with chronic undifferentiated arthritis (UA: n = 177) were analyzed. The protocol included 5 visits during the two-year follow-up period and at each visit, the following data were collected and entered into an electronic database: clinical and demographic information; disease duration at the beginning of follow-up; Disease Activity Score of 28 tender and swollen joint counts (DAS28) [13]; global disease activity on a 100-mm visual analogue scale assessed by both the patient and the physician; the score from the Health Assessment Questionnaire (HAQ; Spanish version) [14]; and the results of laboratory tests including the erythrocyte sedimentation rate (ESR), C-reactive protein (CRP), rheumatoid factor (RF, assessed by nephelometry; positive >20 IU/ml), and anti-citrullinated peptide antibody (ACPA, measured by enzyme immunoassay: Euro-Diagnostica Immunoscan RA; positive >50 IU/ml).

**Figure 2:**
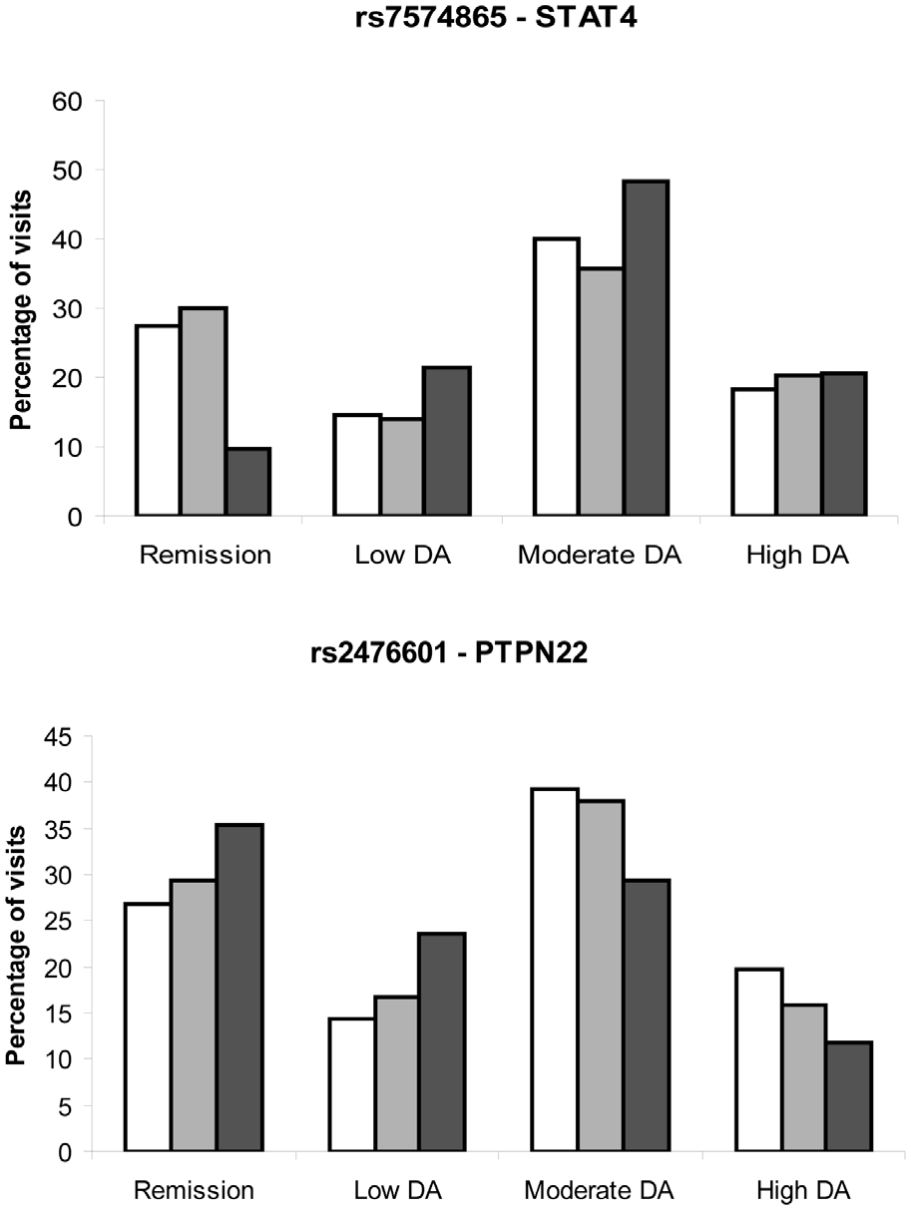
Effect of the rs7574865 and rs2476601 polymorphisms in *STAT4* and *PTPN22* on the level of disease activity in patients with early arthritis. A) STAT4 genotypes: GG, white bars; GT, gray bars; TT, black bars. B) PTPN22 genotypes: CC, white bars; CT, gray bars; TT, black bars. Data represent the proportion of visits in which remission, or low, moderate or high disease activity was recorded in the patients of each genotype. This figure illustrates the findings presented in Table 3, in which the statistical significance of the differences in disease activity associated with the genotype of each polymorphism is shown.

**Table 3:** Effect of the presence of the shared epitope and the minor rs7574865 allele of *STAT4* or rs2476601 in *PTPN22* on the level of disease activity.

	β Coeff. (95% CI)	p value
Age (years)		
<45	Ref.	
45–65	0.64 (0.42–0.86)	<0.001
>65	0.70 (0.47–0.94)	<0.001
Gender		
Man	Ref.	
Woman	0.65 (0.44–0.86)	<0.001
Diagnosis		
RA	Ref.	
UA	−0.51 (−0.79– −0.23)	<0.001
Positive ACPA	0.32 (0.13–0.51)	0.001
Shared epitope	NS	NS
*STAT4* (rs7574865)		
GG	Ref.	
GT	−0.02 (−0.21–0.16)	0.785
TT	0.56 (0.01–0.83)	0.002
*PTPN22* (rs2476601)		
CC	Ref	
CT	−0.29 (−0.54– −0.04)	0.025
TT	−0.55 (−1.44–0.33)	0.221
Hospital		
Center 1	Ref.	
Center 2	−0.25 (−0.45– −0.06)	0.01
Cutpoints		
Remission/Low disease activity	−0.10 (−0.40–0.20)	–
Low/Moderate disease activity	0.55 (0.24–0.85)	–
Moderate/High disease activity	2.34 (2.02–2.66)	–

DAS28, 28-joint Disease Activity Score; HAQ, Health Assessment Questionnaire; Coeff., coefficient; Ref., reference; RA, rheumatoid arthritis; UA, undifferentiated arthritis; ACPA, anti-citrullinated peptide antibodies; NI, not included; NS, non-significant.

### Description of the Procedures

#### Genotyping

DNA was obtained from peripheral blood using standard methods, and the samples were genotyped for the *PTPN22* rs2476601 and the *STAT4* rs7574865 genetic variants using a predesigned SNP Genotyping Assay (Applied Biosystems, Part numbers: C_16021387_20 and C_29882391_10, respectively: Foster City, California, USA). Polymerase chain reactions (PCRs) were carried out according to the manufacturer’s instructions and after PCR, the genotype of each sample was automatically attributed by measuring allele-specific fluorescence on an ABI Prism 7900 Sequence Detection System using SDS version 2.3 (Applied Biosystems). Duplicate samples and negative controls were included to verify the genotyping accuracy.

**Figure 3:**
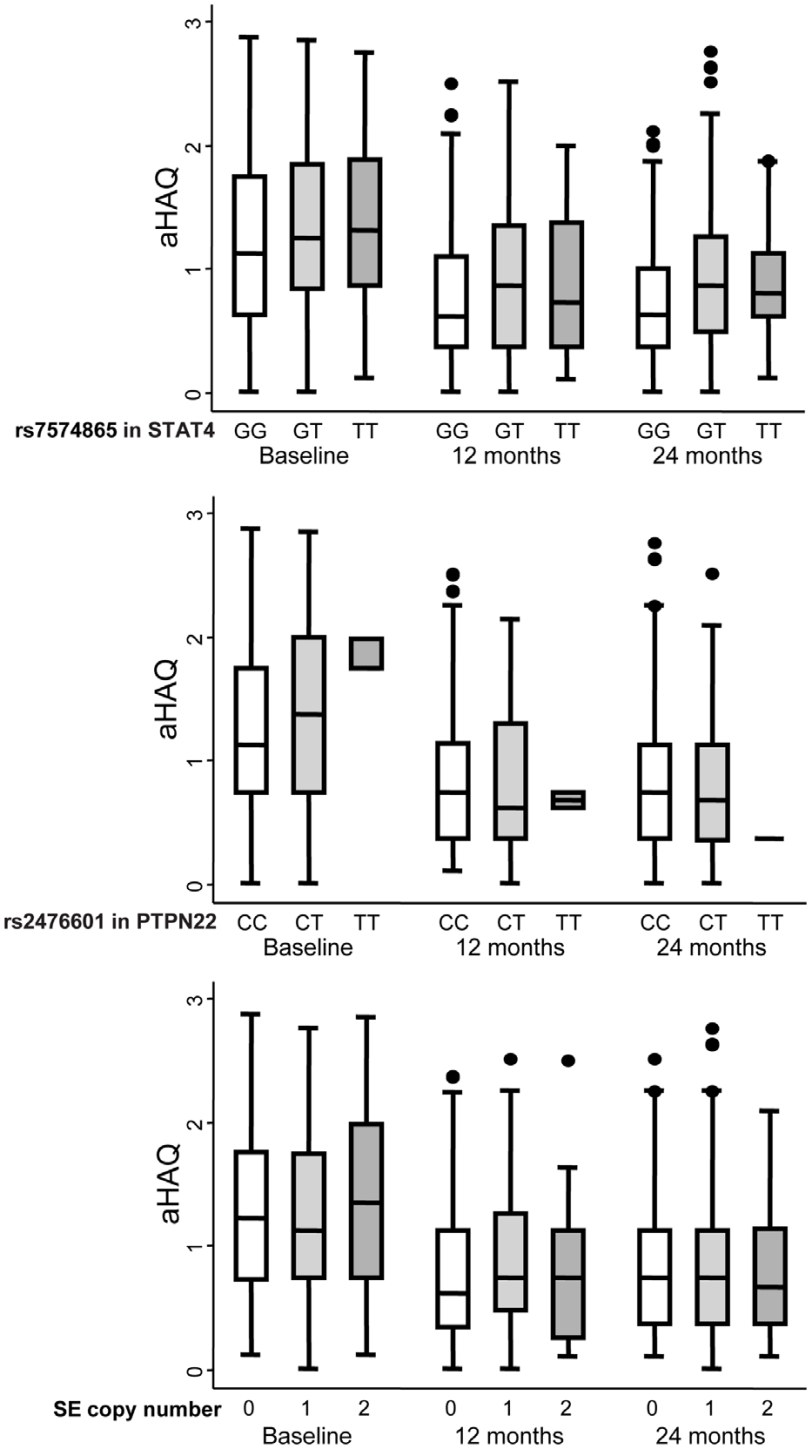
Patients with early arthritis who are homozygous for the minor rs7574865 allele of *STAT4* exhibit increased disability during follow-up. The data are categorized according to the rs7574865 genotype of *STAT4* (upper panel), the rs2476601 genotype of *PTPN22* (middle panel), and the number of copies of the shared epitope (SE; lower panel). The data are presented as the interquartile range (p75, upper edge of the box; p25, lower edge; p50, midline), the p95 (line above box) and p5 (line below the box) of the HAQ adjusted for gender and age (aHAQ). Dots represent outliers.

*HLA-DRB1* was genotyped using a reverse dot-blot kit with sequence-specific oligonucleotide probes (Dynal RELI™ SSO *HLA-DRB1* typing kit: Dynal Biotech, Bromborough, UK). When necessary, high-resolution typing of *HLA-DRB1_03* samples was performed using Dynal AllSet™ SSP DRB1_03.

### Statistical Analysis

Normally distributed quantitative variables were represented as the mean (± standard deviation: SD), while non-normally distributed variables were represented as the median and interquartile range (IQR). Qualitative variables were described using an estimation of the proportions. Variables with a normal distribution were analyzed by the *t*-test, while the Mann Whitney or Kruskal-Wallis tests were used for variables with a non-normal distribution. A χ^2^ or Fisheŕs exact test was used to compare categorical variables.

Only patients whose DAS28 or HAQ scores were available on at least two visits were considered for analysis. In addition, the genotype of *HLA-DRB1*, *PTPN22* or *STAT4* was not available (lack of DNA sample or undetermined) in 5%, 9% and 8.7% of cases, respectively. Therefore, we used data from 1,718 visits (449 patients; 3.8 visits/patient) to analyze disease activity and data from 1,813 visits (475 patients; 3.8 visits/patient) to analyze disability. To identify factors that influenced disease activity (DAS28 as the dependent variable) and disability (HAQ as the dependent variable) during the follow-up, we fitted two population-averaged models by generalized linear models nested by patient and visit using the *xtgee* command of Stata 10.1 for Windows (StataCorp LP, College Station, Texas, USA). The population-averaged generalized estimating equations were first modeled by adding all variables with a *p* value <0.15 in the bivariate analysis. The final models were constructed using quasi-likelihood estimation based on the independence model information criterion [15] and Wald tests, removing all variables with *p*>0.15.

In addition, to confirm the results obtained for disease activity we generated an ordered logistic model using the *ologit* command of Stata. The dependent variable was the disease activity level, using the cut-off points for DAS28 proposed by Prevoo et al. (<2.6, remission; 2.6 to 3.2, low disease activity; 3.2 to 5.1, moderate disease activity; >5.1 high disease activity [13]). The analysis was modeled as described above for *xtgee*, with remission considered 0 and low, moderate and high disease activity represented as 1, 2 and 3. The ordered logistic analysis estimates cut-off points that aid the interpretation of the coefficients for each independent variable according to the levels of the dependent variable.

## Results

### Population

At baseline, patients with definitive RA exhibited increased disease activity and disability, and higher acute phase reactant values than patients with UA (Table 1). Similarly, the percentages of RA patients positive for ACPA, RF and the SE were significantly higher than among UA patients (Table 1). However, UA patients were younger and tended to exhibit shorter disease duration than RA patients (Table 1). No significant differences were observed between the two subgroups in the distribution of the polymorphisms in *STAT4* and *PTPN22* studied (Table 1).

### The STAT4 rs7574865 Polymorphism is Associated with the Evolution of Disease Activity in Early Arthritis Patients

The DAS28 values were significantly higher in elderly and female patients, those fulfilling the RA criteria, those with positive ACPA, and in patients from one of the centers (Table 2). After adjusting for these confounding variables, patients who were homozygous for the minor allele rs7574865 in *STAT4* displayed significantly higher DAS28 values (β coefficient 0.42 [95% confidence interval: 0.01–0.83], p = 0.044: Table 2 and Figure 1), whereas the presence of a SE copy had no significant effect on disease activity. By contrast, the presence of the minor allele of rs2476601 in *PTPN22* was associated dose-dependently with lower DAS28 values when compared with CC homozygous patients, although this effect was not significant for the TT genotype due to the low number of cases in our population (β coefficient for CT genotype −0.27 [−0.56– −0.01], p = 0.042; TT genotype −0.68 [−1.64– −0.27], p = 0.162: Figure 1 and Table 2).

To confirm our findings and obtain additional information, we analyzed the effects of these variables when disease activity is categorized as remission, or as low, moderate or high disease activity. Patients with the TT genotype of the rs7574865 allele in *STAT4* displayed greater disease activity than heterozygous or GG genotype patients (Figure 2A). By contrast, the presence of a T allele of rs2476601 in *PTPN22* was associated with more visits at weaker disease activity or remission (Figure 2B). Using this approach, multivariate analysis revealed a stronger association between the ACPA and TT genotype of rs7574865 in *STAT4* and increased levels of disease activity, without significant changes in the contribution of the other independent variables when compared with the previous model (gender, age, *etc.*: Table 3). In addition, the cut-off points obtained in the ordered logistic regression model (rows at the bottom of Table 3) suggest that variables with positive coefficients, such as ACPA, gender or TT genotype of rs7574865 in *STAT4,* increased disease activity to moderate or high levels, while those with negative coefficients, such as the minor allele of rs2476601 in *PTPN22*, decreased the disease activity to the level of remission.

### The Effect of the STAT4 rs7574865 Polymorphism on Disability Reported by Early Arthritis Patients

As expected, disability was significantly correlated with DAS28 values. Moreover, female gender and older age were associated with significantly higher HAQ values. After adjustment for these confounding variables, we detected a significant dose-dependent effect of the rs7574865 T allele of *STAT4* on the HAQ value (Table 2 and Figure 3). Neither the presence of the SE, or of the *PTPN22* rs2476601 minor allele, contributed to disability in our population.

## Discussion

To the best of our knowledge, this is the first study demonstrating the influence of the *STAT4* polymorphism rs7574865 on disease course and disability in EA. *STAT4* is a member of the *STAT* family and it is implicated in the signals mediated by interleukin (IL) 12, IL-23 and type 1 interferons [16]. Although the mechanism by which the minor allele of rs7574865 contributes to the rupture of self-tolerance is not well understood, it is thought to involve dysregulation of helper T cell differentiation towards T_H_1 and/or T_H_17 phenotypes [16]. Kariuki et al reported that lupus patients with the minor rs7574865 allele of *STAT4* exhibit lower levels of serum interferon-α but a comparatively stronger biological responses to this cytokine [17]. Moreover, the minor allele of rs7574865 has also been associated with severe disease in patients with systemic lupus erythematosus [18]. Therefore, the more intense disease activity observed in patients who were homozygous for the rs7574865 T allele may be associated with increased sensitivity to IL-12, IL-23 or interferons.

By contrast, the presence of the minor allele of rs2476601 in *PTPN22* was associated with a trend towards diminished disease activity. The functional effect of this polymorphism is open to debate, although most studies suggest that the allele promotes a gain of function and hence, an increased threshold for T cell receptor (TCR) signaling that may lead to defective deletion of autoreactive clones in the thymus [3], [19]. Our results could support this hypothesis, suggesting that once self-tolerance is broken in patients with the rs2476601 T allele, autoreactive lymphocytes are activated less efficiently through TCR.

Surprisingly, the presence of the SE was not associated with increased disease activity or disability, probably due to the inclusion of ACPA in the statistical models. However, excluding this variable revealed no significant association between the presence of the SE and a worse outcome (data not shown). Although unexpected, there may be several explanations for this finding: a) a proportion of SE carriers were non-smokers and thus, they did not develop ACPA; b) some patients may be heterozygous carriers of SE and carry protective DRB1 alleles; or c) SE may be less important in RA patients from southern Europe than northern Europe. Taken together, these findings suggest that ACPA is a better marker of disease severity than SE. As with *PTPN22*, specific genetic factors may confer a risk of developing the disease, and while some may only modify disease severity, others may be involved in both processes.

Few studies have evaluated the effects of genetic markers that confer a risk of developing RA on disease activity and disability. In one study, the influence of 5 susceptibility genes on disease severity was analyzed, including that of DRB1, STAT4 and PTPN22 [11]. Only a weak association was reported between TRAF1/C5 and disease severity, as assessed by swollen joint counts, with no association of STAT4 or the remaining genes. The conflicting findings of this and our study may be due to the differences in study design. As opposed to the cross-sectional cohort study carried out previously, we performed a longitudinal study with almost 4 observations per patient, providing a more detailed picture of disease activity and disability. Our study involved patients from 2 clinical centers, a confounding variable for which the analysis of disease activity was adjusted. This is particularly important given the poor reproducibility of tender and swollen joint counts [20]. Furthermore, the study of Morgan and coworkers only adjusted the analysis for disease duration, which was longer than in our cohort. However, probably the most significant factor contributing to the divergent findings is the genetic background of the populations analyzed, as TRAF1/C5 and SE had a stronger influence on RA in the British population, and STAT4 in the Spanish population.

In summary, our data suggest that homozygosity for the *STAT4* rs7574865 minor allele is associated with a poorer disease course and greater disability than homozygosity for the common allele of this variant. We propose that the rs7574865 polymorphism may be used as a molecular tool to identify patients that may benefit from early aggressive treatment.
